# What Augmented Physical Activity and Empowerment Can Bring to Patients Receiving Total Knee Replacement: Content, Implementation, and Comparative Effectiveness of a New Function-Tailored Care Pathway in a Routine Care Setting

**DOI:** 10.1155/2015/745864

**Published:** 2015-04-16

**Authors:** G. van der Sluis, R. A. Goldbohm, R. Bimmel, F. Galindo Garre, J. Elings, T. J. Hoogeboom, N. L. U. van Meeteren

**Affiliations:** ^1^Department of Physiotherapy, Nij Smellinghe Hospital, Compagnonsplein 1, 9202 NN Drachten, Netherlands; ^2^TNO, Healthy for Life, Leiden, Netherlands; ^3^Department of Orthopedics and Traumatology, Nij Smellinghe Hospital Drachten, Drachten, Netherlands; ^4^Center for Biostatistics, VU University Amsterdam, Amsterdam, Netherlands; ^5^Department for Physiotherapy, Diakonessenhuis Hospital Utrecht, Utrecht, Netherlands; ^6^Department of Epidemiology, Maastricht University Medical Centre Maastricht, Netherlands; ^7^CAPHRI School for Public Health and Primary Care, Maastricht, Netherlands; ^8^Center for Care Technology Research, Maastricht, Netherlands

## Abstract

*Background*. In the routine setting of the 20-bed orthopaedic ward of a regional hospital in Netherlands, we developed, implemented, and evaluated a new, function-tailored perioperative care pathway for patients receiving total knee replacement (TKR), aimed at faster functional recovery by reduction of inactivity and stimulation of self-efficacy of the patients. *Methods*. To assess effectiveness, we compared, using prospectively collected data from medical files, patient groups before (*n* = 127) and after (*n* = 108) introduction of the new care pathway with respect to time to recovery of physical functioning during hospitalisation (five milestones), length of hospital stay (LoS), referrals to an inpatient rehabilitation facility, and readmissions. Multivariable regression was used to adjust results for differences between the two groups in preoperatively assessed risk factors for delayed recovery. *Results*. Comparison of patient groups before (*n* = 127) and after (*n* = 108) introduction of the tailored care pathway showed that the tailored rehabilitation pathway decreased the time to recovery of physical functioning (from 4.5 to 4.1 days, *P* < 0.05), the mean LoS (from 5.2 days to 4.2 days, *P* < 0.01). *Conclusion*. We demonstrated that the introduction of a function-tailored care pathway shortens the hospital stay and accelerates the recovery of physical functioning.

## 1. Background

Osteoarthritis is the most common joint disorder of older people and is the most common indication for total knee replacement (TKR) [1]. The success of arthroplasty depends not only on effective surgery but also on adequate postoperative rehabilitation of the patient [2]. Although total joint replacement is successful in the majority of patients, it is associated with serious medical risks, diminished functional capacity, and potentially persistent pain [3–5]. As with all medical interventions, steps should be taken to minimise the risk of serious adverse medical events. For example, bed rest and inactivity are harmful for patients, in particular for elderly or frail patients, in terms of disability and loss of functional capacity [6]. Brown et al. (2009) showed that older patients are inactive 83% of the time they are hospitalised, about 20 hours a day [7]. Current attitudes to hospitalisation are likely to foster inactivity and to reinforce passive coping strategies among patients.

There is a plethora of evidence demonstrating that, to counterbalance prolonged hospitalisation and physical inactivity, therapists should initiate postoperative rehabilitation as soon as possible after TKR surgery [8–10]. To ensure a discharge that is both timely and adequate, both therapist and patient need to monitor the patient's progress of achieving functional independence, even before the patient is admitted to the hospital [11]. Ideally, such is clearly described in an interdisciplinary clinical care pathway [12] and the patient is stimulated to self-manage their recovery [13].

Along these lines, we developed and implemented a new, function-tailored care pathway to rehabilitate TKR patients. The goals of the pathway are to reduce the bed rest period to 4 hours postoperatively, to set realistic short-term rehabilitation goals based on relevant functional milestones, and to improve the patient's self-efficacy by stimulating an active coping attitude and environment (staff and infrastructure) during hospitalisation. Our pathway is distinct from other care pathways, as its focus lies on the functional ability of patients undergoing surgery rather than on successful surgery and postoperative care of patients with a disease [14].

In this paper, we describe the content and implementation of the function-tailored care pathway. Our aim was to study whether a function-tailored care pathway compared to the usual care situation before its introduction was able to reduce the time needed to achieve functional independence during hospital stay and length of in-hospital stay.

## 2. Patients and Methods

### 2.1. Setting

Nij Smellinghe, Drachten, Netherlands, is a small regional hospital with 320 beds. The orthopaedic ward has 20 clinical beds and 150 primary TKR are performed annually. The healthcare team in charge of the treatment of patients scheduled for elective TKR and other major elective and traumatic orthopaedic surgeries consists of four orthopaedic surgeons, four nurse practitioners, four physiotherapists, and thirty-five nurses.

### 2.2. Usual Care for Elective TKR before Implementation of the New, Tailored Care Pathway

The key elements of the usual care pathway were as follows. Two weeks prior to surgery patients were informed on the operation and hospital admission procedure during a group session. Preoperative screening was performed by an anaesthesiologist. All patients were admitted one day before surgery. The relevant information on the patient was collected during the preoperative screening and on the day of admission (e.g., interview about functional and social status, blood samples, relevant anaesthetic information, etc.). During the hospital stay, the patient was rehabilitated according to a protocol organised as a time table, treating every patient in the same manner: “one size fits all.” The inpatient rehabilitation programme consisted of training of transfers, ambulation, and stair climbing.

According to this protocol, if no complications arose, each TKR patient was to be mobilised 24 hours after surgery and discharged home 4 days after the day of surgery. If availability of informal care was insufficient to allow safe return home, patients were discharged to a rehabilitation centre 4 days after surgery.

This usual care protocol was adopted from joint care principles, which is a standard care protocol for patients undergoing total hip and total knee surgery.

### 2.3. The New Function-Tailored Care Pathway for Elective TKR

The elements of the newly developed function-tailored care pathway were introduction of (1) preoperative screening of physical functioning, (2) postoperative monitoring of recovery of physical functioning, (3) fast track tailored rehabilitation, (4) communication with the patient to improve self-efficacy, and (5) improvement of collaboration, communication, and knowledge of the health professionals involved. The elements are described in more detail below.

#### 2.3.1. Preoperative Screening of Physical Functioning

Physical functioning of patients was screened preoperatively by a physiotherapist at the same session as the anaesthesiologist carried out the preoperative medical screen. Physical functioning was assessed with the 6-minute walk test (6MWT) [15], the Timed Up and Go test (TUG) [16], and the De Morton Mobility Index [17], as well as several questionnaires, that is, the Western Ontario and McMaster Universities Arthritis Index (WOMAC) [18], pain perception by use of the visual analogue scale (VAS) [19], and the Identification of Seniors at Risk (ISAR). Validated instruments were used for the assessments and all physiotherapists at the orthopaedic ward were trained to apply and record these according to the guidelines on structured forms, which were included in the patient's medical file.

#### 2.3.2. Assessment of Postoperative Functional Recovery during Hospital Stay

Measurement of functional milestones enabled the physiotherapist to assess whether and when a patient can function independently and allowed tailoring of treatment goals to individual patients [20, 21]. The MILAS (Modified Iowa Levels of Assistance Scale) was used daily to monitor the recovery of physical function. The data were collected according to the guidelines for the instruments, entered on structured forms, and included in the patient's medical file.

#### 2.3.3. Fast Track Tailored Rehabilitation

In order to minimise postoperative immobilisation, we implemented “fast track” rehabilitation principles [8–10]. From the day of surgery until discharge the patient received physiotherapy tailored to the patient's capacity to regain relevant functional activities. This was organised as follows. Patients were allowed to stay in bed for 4 hours after surgery. From day 0 (day of surgery) until discharge all patients received physiotherapy at least twice daily, including weekends. If necessary to achieve treatment goals, patients were treated more frequently. The frequency and content of the rehabilitation depended on the specific problems experienced by the patient while trying to achieve relevant functional milestones (e.g., muscle weakness, coping strategies, fear avoidance, motor learning capacities, etc.). The postoperative physiotherapy consisted of (1) exercises to improve the range of motion of the knee joint (starting on the day of surgery); (2) muscle exercises in sitting and standing position to regain muscle feeling/power (starting on day of surgery); (3) exercising the functional milestones to retrieve functional independence (assessed daily with the MILAS, starting on day of surgery).

#### 2.3.4. Communication with the Patient to Improve Self-Efficacy

As the rationale behind the function-tailored clinical pathway was to increase patients' self-efficacy, the primary purpose of patient communication was to manage and influence patient expectations. We organised a group session two weeks before surgery, as part of the preoperative screening procedure, to inform patients about the procedures and expectations around surgery and hospitalisation and to answer questions. During hospitalisation, communication with the patient was directed at identifying patients' needs to function independently in his home environment and to achieve shared decisions between patient and health professional. A second group session was held on the second postoperative day, during which patients were involved in rehabilitation decisions after discharge. In both sessions and during individual contacts, emphasis was on encouraging patients to manage their own recovery and to have confidence in their own abilities [22].

#### 2.3.5. Improvement of Collaboration, Communication, and Knowledge of the Health Professionals Involved

The overall aim was to improve the expertise of the interdisciplinary team with respect to physical functioning and rehabilitation principles and to improve collaboration and communication within the team. The process was guided by three principles: (I) value for the patient, (II) patient specific problems (e.g., preoperative functional status, coping strategies, context, etc.) which restrict involvement of each specific health profession, and (III) focus on (recovery of) physical functioning. In this new situation, the nursing staff, under the supervision of a physiotherapist, was charged with the mobilisation of patients [22].

Moreover, consensus was reached on discharge criteria, namely, when (1) the orthopaedic surgeon and anaesthesiologist had completed their medical treatment; (2) functional recovery (functional milestones) was adequate for the discharge destination; and (3) adequate care could be provided in time at the discharge destination.

To ensure a successful implementation of these elements, we used an implementation strategy developed by Grol and Grimshaw [23]. This strategy comprises four steps, with its specific interventions, in order to introduce a change in healthcare procedures: (1) orientation, (2) insight, (3) acceptation, and (4) changing.

We carried out these four steps for each specific element of the new care pathway. In Table 1, the details of the complete implementation strategy and time schedule are depicted using a pat-plot [24]. The first two elements of the new care pathway were implemented in April 2009, to gain insight into the characteristics of the TKR patient population, including their functional status during the perioperative period. The remaining elements, aimed at changing the delivered treatment and care, were subsequently introduced in September 2010.

### 2.4. Design and Patients

To evaluate the effectiveness of the new function-tailored care pathway, we used an observational cohort study design, comparing preoperative characteristics prospectively collected during the screening and outcome measures of the TKR patient groups taken in for surgery before and after introduction of the third, fourth, and fifth components of the new care pathway. Data were anonymously retrieved from the medical files of all 235 patients, except one, who underwent elective primary TKR between 1 April 2009 and 1 November 2011 (*n* = 127 “usual” care, data from April 2009 to September 2010; *n* = 108 function-tailored care, data from October 2010 to November 2011). As one medical file could not be retrieved at the time of data extraction, this patient was excluded. According to Dutch law review by a medical ethical committee was not required for this type of research.

### 2.5. Outcome Measurements

To assess effectiveness of the function-tailored care pathway we measured (1) recovery of physical functioning, defined as the time (in days) between the end of surgery and the day when physical functioning was considered regained according to MILAS (i.e., score of ≤6); (2) hospital LoS, defined as the time (in days) between the day of surgery and hospital discharge; (3) the patient's discharge destination; and (4) readmission within 12 weeks of discharge. The MILAS is a modified version of the Iowa Levels of Assistance Scale (ILAS), which assesses the capability of patients to perform safely four activities of daily life (namely, supine to sit, sit to stand, walking, and stair climbing) and rates the amount of assistance needed. The MILAS adds a fifth activity, namely, the transfer from sit to supine [25]. Scores range from 0 to 30, with scores of six or lower being considered to reflect recovery of physical function. As many patients were already discharged before they were tested for their ability to climb stairs, the cut-off was set before the last MILAS milestone (i.e., stair climbing) was achieved.

To assess patient satisfaction, we also reviewed the returned questionnaires that are routinely sent out to patients on the orthopaedic ward (mainly for total hip and knee replacements and hip fractures) on the day of discharge.

Furthermore, we carried out a semistructured interview with representatives of the involved healthcare professionals (mostly two per discipline). For this semistructured interview we used the method described by Baarda et al. The interviewer (GvdS) developed and applied an “interview guide,” that is, a list of questions and topics that need to be covered during the conversation, in a particular order. We mostly followed this guide but followed topical trajectories in the conversation that deviated from the guide when we thought this was appropriate. Each interview was transcribed verbatim and the transcripts were coded and analysed [26]. Because this qualitative research was not the main focus of this study, only a summary of important findings will be shown in the result section.

### 2.6. Statistical Analysis

Descriptive statistics were computed for the demographic, preoperative, and postoperative variables. Differences between the two patient groups were tested with independent *t*- and chi-squared tests. As in all epidemiological studies, investigation of and adjustment for confounders are an important part of the data-analysis. Based on previous research using more variables available from the same dataset, we identified a number of predictors of the outcome, in particular functional recovery. These predictors are considered potential confounders and become true confounders that have to be adjusted for if their distribution differs between the two patient groups. We used multivariable regression techniques to adjust for these differences in relevant preoperative characteristics between the two care groups. First, we used a tobit regression model to test whether the tailored care pathway was effective in reducing the number of days to recovery of physical functioning. We used this model because we did not have information on recovery of physical functioning after day 7; all patients with a recovery longer than 7 days were assumed to be censored. Second, an analysis of variance (ANOVA) was used to test whether the function-tailored care pathway reduced LoS. This variable was not normally distributed and thus data were logarithmically transformed to meet the model assumptions. Lastly, a logistic regression model was used to test whether the proportion of people going to a rehabilitation clinic changed after implementation of the function-tailored care pathway. To account for any differences in the risk of prolonged recovery between the two groups of patients who underwent TKR before and after implementation of the function-tailored care pathway in September 2010, determinants of this risk, namely, age, body mass index (BMI), TUG, and ISAR (manuscript in preparation), were included in the model [27]. Statistical significance was set at *P* < 0.05 (two-sided). With 110 patients per group, we were able to detect a small to medium effect size (Cohen's *d* = 0.4) with 80% power (alpha of 0.05, two-sided). The statistical package STATA version 11.2 (StataCorp. 2009, Statistical Software: Release 11.2. College Station, TX: Stata Corporation) was used for the analyses.

## 3. Results

### 3.1. Comparative Effectiveness

The preoperative characteristics and postoperative outcomes of the patients who underwent TKR before and after the introduction of tailored care pathway are presented in Table 2.

No patients were excluded, except for one patient from whom the medical record could not be found at the time of data extraction. The two groups differed in terms of recovery (time to MILAS ≤ 6), LoS, and referral to inpatient rehabilitation facilities, but also in some of the determinants of risk of delayed recovery, that is, sex, age, BMI, TUG, and ISAR. The patients who received “usual” care were older (mean 71 versus 70 years, resp.) and took longer to perform the TUG (13.1 versus 11.9 seconds, resp.); the mean BMI was higher in the tailored care group (32 versus 30 kg/m^2^, resp.).

Data for recovery of physical functioning after surgery, as assessed with the MILAS, were available for 79% of the patients (76% and 83% in the “usual” and tailored care groups, resp.). The main reason for missing data was referral of not fully recovered patients to inpatient rehabilitation facilities. The patients in the tailored care group attained a MILAS sum score of six or less 0.5 days earlier than the patients in the usual care group (*P* = 0.02) (Table 3).

Moreover, the mean LoS was also shorter in the tailored care group (Table 4), with 87% (exp(−0.14) = 0.87) decrease in the geometric mean of LoS. Without log transformation of LoS, the mean decrease amounted to 1 day (data not shown).

The tailored care pathway was not associated with a change in the probability of discharge to a rehabilitation facility (odds ratio 0.64, 95% CI 0.32–1.26). Lastly, there were 7 (5.5%) and 8 (7.4%) readmissions in the usual and tailored care groups, respectively (nonsignificant) (Table 5).

Differences between the two care groups in recovery of physical functioning and LoS did not vary significantly across categories of the baseline variables (age, gender, BMI, TUG, and ISAR, data not shown). The returned patient questionnaires (response rate 23%) showed no difference in patient satisfaction between the two care pathways (data not shown).

### 3.2. Acceptance by the Staff

Analysis of the semistructured interviews for the health professionals revealed that orthopaedic surgeons were surprised that screening patients' physical functioning before surgery could contribute to a tailored rehabilitation process and therefore more efficient discharge policy of the patient. They also found that setting functional goals considerably improved the care and recovery of patients who had undergone TKR. Nurse practitioners mentioned similar aspects and added that they experienced a greater responsibility for the perioperative care and discharge planning of patients undergoing TKR. Physiotherapists mentioned the increased focus on measurable physical functioning and the interdisciplinary responsibility for patients' functional recovery as most important benefit of the new care pathway. The nursing staff was positive about the extension of their job description with basic tasks aimed at patient rehabilitation, although some nurses doubted their ability to perform these tasks.

## 4. Discussion

We found that changing from “one size fits all” perioperative care to tailored care using functional goals for patients undergoing TKR accelerated the recovery of physical functioning on average by 0.5 days and shortened the LoS. The shortened LoS was not achieved at the expense of significantly more referrals to inpatient rehabilitation facilities, significantly more readmissions, or patient satisfaction. In addition, healthcare professionals were positive about the tailored care pathway, feeling that it improved patient care.

This study is performed in regular practice, which means that there is no selection of patients. This is an important strength of this study which means that one can implement such innovative pathway developments in regular practice.

Another strength of this study is that the data used in the analyses, with the exception of the interviews with the health professionals, came from data collected as part of routine care. The availability of preoperative baseline information, prospectively collected with validated instruments according to scientific standards at the preoperative functional screening, and availability of well-monitored outcome information made it possible to apply a valid epidemiological study design. This shows that well-kept, up-to-date patient records that include relevant baseline data can form a source of information to monitor the effectiveness of changes in routine medical care and management.

Our study had several limitations.

- Postoperative complications that did not result in hospital readmission and outcomes after hospital discharge (in particular functioning and quality of life) were not monitored, although this piece of information is important for assessing the longer-term effectiveness of the new tailored care pathway [28].
- We formally monitored compliance with two of the key elements of the care pathway, that is, preoperative screening and postoperative milestones, and monitored the last key element (i.e., team mission) qualitatively. Unfortunately, we did not extract data from the medical file on elapsed time between completion of surgery and start of mobilisation (key element 3), nor did we have sufficient data on compliance with key element 4, that is, enhancement of self-efficacy.
- We evaluated patient satisfaction using the standard patient evaluation questionnaire that is routinely administered on the orthopaedic ward on the day of discharge, but the response rate was low because not all patients received the questionnaire. Patient satisfaction should be reevaluated in a future study.
- Although we were able to adjust the differences in outcome between the two patient groups for the most important confounders, we cannot exclude some residual confounding by unmeasured factors.

Because of the increased awareness that a successful functional outcome of TKR and a shorter LoS are achievable, health care professionals are interested in clinical pathways with fast track approaches. Our new care pathway for TKR patients focused only on the early and more intensive mobilisation approach.

A challenge for the near future is to investigate if and how patients at risk for a prolonged functional recovery would benefit, with faster recovery, from state-of-the-art perioperative physiotherapy, anaesthesiology, and nutritional interventions [8, 25, 29]. As effect of these interventions, we expect that a larger number of patients could be mobilized according to the fast track rehabilitation protocol [8–10, 29]. This might be particularly relevant to the older and fragile patients, as long hospitalisation is especially harmful to them [6].

In conclusion, we demonstrated that the introduction of a new TKR care pathway, focused more systematically on functional recovery of patients, speeded recovery of physical functioning and shortened the hospital stay. The shortened LoS was not achieved at the expense of significantly more referrals to inpatient rehabilitation facilities, significantly more readmissions, or patient satisfaction. Moreover, the staff enjoyed working in an interdisciplinary team, sharing goals and commitments and gaining additional insights into the healthcare process. Furthermore we showed that evaluation of innovations in health care is feasible in a routine care setting and should be encouraged.

## Figures and Tables

**Table 1:** Content and timeline of the implementation process of a new, tailored care pathway for TKR.

Timeline	Physical therapist	Nursing staff	Orthopeadic surgeon	Hospital management
January 2009	A^*^			
February 2009	B^*^			
March 2009	C^*^	D^*^	D^*^	
April 2009	A^#^			
January 2010				B^#^
June 2010	E^*^	E^*^		
September 2010	C^#^ D^#^	C^#^ D^#^	C^#^ D^#^	
November 2010	F^*^	F^*^		
December 2010				G^*^
January 2011	H^*^	H^*^		
March 2011	I^*^	I^*^		
June 2011	I^*^	I^*^		
September 2011	I^*^	I^*^		

Components (c) with their aims (A)

A^*^ **C**: information seminar about important preoperative risk stratification **A**: to improve evidence based practice for physical therapy

B^*^ C: training session; preoperative risk stratification measurement instruments **A**: to improve risk stratification expertise

C^*^ C: discussion of patient casuistry A: to improve patient specific care

D^*^ C: information session about adapted physical therapy strategy A: to improve knowledge and communication

E^*^ C: teaching/schooling about importance of an active approach in functional recovery after surgery

F^*^ C: rehearsal meeting about importance of an active approach in functional recovery after surgery

G^*^ C: informing about the adapted physical therapy strategy and its results

H^*^ C: establishment of a task force concerning the active approach in functional recovery after total joint replacement

I^*^ C: evaluation meeting task force

A^#^ C: implementing preoperative functional screening as usual care

B^#^ C: embedding functional screening in the standard preoperative hospital screening

C^#^ C: introducing fast track rehabilitation principles

D^#^ C: functional recovery is leading in hospital admission

**Table 2:** Characteristics and outcomes of patients who had total knee replacement surgery before and after introduction of a function-tailored care pathway.

	Before (*n* = 127)				After (*n* = 108)				Total (*n* = 235)			
	*n*	Mean/%	Median	95% CI	*n*	Mean/%	Median	95% CI	*n*	Mean/%	Median	95% CI
Preoperative characteristics												
Sex (% men)	127	26.0			108	29.6			235	27.7		
Age (years)	127	71.1	72	69.6–72.6	108	70.4	71	68.6–72.2	235	70.8	72	69.7–71.9
BMI (kg/m^2^)	127	29.9	29.1	28.9–30.8	108	32.2	29.0	29.5–34.9	235	31.0	29.1	29.6–32.3
ISAR	112^1^	1.2	1	1.0–1.5	108	1.1	1	0.8–1.3	220	1.1	1	1.0–1.3
Timed Up and Go test (s)	127	13.1	10.4	11.2–14.9	108	11.9	10.2	10.7–13.2	235	12.5	10.3	11.4–13.7
Outcomes												
Time to MILAS ≤6 (days)	96^2^	4.5	4	4.3–4.7	90^2^	4.1^*^	4	4.0–4.3	186	4.3	4	4.2–4.4
Length of stay (days)	127	5.2	5	4.9–5.5	108	4.2^*^	4	4.1–4.4	235	4.8	4	4.6–5.0
To rehabilitation clinic (% yes)	40	31.5			27	25.0			67	28.5		
Readmission (% yes)	7	5.5			8	7.4			15	6.4		

CI: confidence interval; BMI: body mass index; ISAR: Identification of Seniors at Risk; MILAS: Modified Iowa Levels of Assistance Scale. ^1^Missing cases due to delayed introduction of ISAR; ^2^missing cases on MILAS due to referral of not fully recovered patients to inpatient rehabilitation facilities; ^*^statistically significant (*P* < 0.05), after versus before.

**Table 3:** Difference in the number of days to functional recovery (according to MILAS ≤6) between TKR patients undergoing surgery before (*n* = 111) and after (*n* = 104) introduction of a function-tailored care pathway, adjusted for preoperative characteristics (multivariable tobit regression analysis).

Independent variables	Coefficient (days)	95% CI	
After versus before	**−0.54** ^*^	**−0.99**	**−0.09**
Age (y)	0.06^**^	0.04	0.09
BMI (kg/m^2^)	0.05^*^	0.01	0.10
ISAR	0.30^*^	0.07	0.54
TUG (s)	0.11^**^	0.06	0.15

BMI: body mass index; ISAR: Identification of Seniors at Risk; TUG: Timed Up and Go test.

^*^ *P* < 0.05, ^**^ *P* < 0.01.

**Table 4:** Difference in length of stay (logarithm days) between TKR patients undergoing surgery before (*n* = 111) and after (*n* = 104) introduction of a function-tailored care pathway, adjusted for preoperative characteristics (multivariable regression analysis).

Independent variables	Coefficient	95% CI	
After versus before	**−0.140** ^**^	**−0.200**	**−0.080**
Age (y)	0.004	−0.000	0.008
BMI (kg/m^2^)	0.005	−0.001	0.011
ISAR	0.084^*^	0.054	0.114
TUG (s)	−0.004	−0.010	0.001

BMI: body mass index; ISAR: Identification of Seniors at Risk; TUG: Timed Up and Go test.

^*^ *P* < 0.05, ^**^ *P* < 0.01.

**Table 5:** Differences in the probability of referral to inpatient rehabilitation facilities between TKR patients undergoing surgery before (*n* = 111) and after (*n* = 104) the introduction of a function-tailored care pathway, adjusted for preoperative characteristics (multivariable logistic regression).

Independent variables	Odds ratio	95% CI	
After versus before	**0.64**	**0.32**	**1.26**
Age (y)	1.12^**^	1.06	1.18
BMI (kg/m^2^)	1.02	0.98	1.05
ISAR	1.07	0.78	1.47
TUG (s)	1.05	0.99	1.12

BMI: body mass index; ISAR: Identification of Seniors at Risk; TUG: Timed Up and Go test.

^**^ *P* < 0.01.
